# The impact of religious festival on roadside livestock traders in urban and peri-urban areas of Yogyakarta, Indonesia

**DOI:** 10.14202/vetworld.2019.1408-1415

**Published:** 2019-09

**Authors:** Alek Ibrahim, I Gede Suparta Budisatria, Rini Widayanti, Wayan Tunas Artama

**Affiliations:** 1 Study Program of Veterinary Science, Faculty of Veterinary Medicine, Universitas Gadjah Mada, Yogyakarta, Indonesia; 2 Department of Animal Production, Faculty of Animal Science, Universitas Gadjah Mada, Yogyakarta, Indonesia; 3 Department of Biochemistry and Molecular Biology, Faculty of Veterinary Medicine, Universitas Gadjah Mada, Yogyakarta, Indonesia

**Keywords:** Eid al-Adha, Indonesia, livestock trade, Muslim festival, roadside seller

## Abstract

**Background and Aim:**

Eid al-Adha is one of the most important festivals celebrated by Muslims in Indonesia. Roadside livestock traders open their stalls during the Eid al-Adha period. This study aimed to investigate the characteristics and behaviors of roadside livestock traders in urban and peri-urban areas in Yogyakarta, Indonesia.

**Materials and Methods:**

In-depth interviews with 36 roadside livestock traders were conducted on August 7-23, 2018 in urban (n=20) and peri-urban (n=16) areas of Yogyakarta. The collected data were analyzed by descriptive and statistical analysis using one-way analysis of variance.

**Results:**

The results indicate that the trading activities of roadside livestock traders in urban areas last longer (p<0.05) than in peri-urban areas. No difference was found in the opening day of stalls, the number of buyers, and trends in animal prices set by roadside livestock traders in urban and peri-urban areas. Most traders sell sheep and goats, buy livestock at the animal market, and only open their stalls during Eid al-Adha. Prices are high in this period, and buyers directly visit the stalls. A significant difference exists in the selling price of livestock between Eid al-Adha and ordinary days (non-festival), and most roadside traders benefit from the Eid al-Adha momentum.

**Conclusion:**

Significant similarities exist among roadside livestock traders during the Eid al-Adha period in urban and peri-urban areas of Yogyakarta, Indonesia. Sheep are more desirable than goats and cattle in this period, and Eid al-Adha generates a high profit for roadside livestock traders.

## Introduction

Eid al-Adha is one of the most important Muslim festivals celebrated worldwide [1]. Muslims traditionally slaughter animals such as sheep, goats, buffalo, cattle, and camels on a sacred day to commemorate the mercy of Allah, who spared Prophet Ibrahim from having to kill his son, Ishmael. Muslims around the world gather on Eid al-Adha to sacrifice their livestock [2]. This observance culminates in the Hajj, and every household who has financial ability sacrifices a male domestic ruminant (such as a yearling ram) in honor of Ibrahim and as a demonstration of obedience to Allah. Three days of celebration and feasting follow Eid al-Adha [3]. In Indonesia, this festival is also known as Idul Adha or Hari Raya Haji. Indonesia is the world’s largest Muslim-majority nation, and Eid al-Adha is one of the largest religious festivals [1]. Sacrificial animals (*kurban*) are slaughtered on the feast of Eid al-Adha (10^th^ Zulhijjah) and the Tasyrik day, which is the 11^th^, 12^th^, and 13^th^ Zulhijjah in the Islamic calendar [4]. The number of livestock slaughtered during Eid al-Adha in 2016 was 1,019,777 heads: 279,211 heads of cattle, 7535 buffaloes, 650,583 goats, and 82,438 heads of sheep [5].

The Eid al-Adha period has a significant impact on the supply of and demand for small ruminants [6]. Two weeks before the Eid al-Adha celebration, some people begin selling livestock on the roadside, although they do not normally sell livestock. These individuals are called roadside livestock sellers or roadside traders [7]. The roadside is chosen for its strategic position to display their livestock. The stalls are usually opened in urban or peri-urban areas. To the best of our knowledge, no previous study examined the characteristics and behavior of roadside traders on religious festival, especially in Indonesia. Such trade activities have come to constitute local wisdom over a period of time and support the need for sacrificial animals, especially in areas dominated by Muslims. Research on the characteristics and patterns of the marketing adopted by livestock traders may help regulate this market and achieve higher collective benefit. The research results can be used as a basis for the implementation of social marketing strategies to improve food safety awareness [1]. Production, procurement, and sales of livestock in the most favorable conditions guarantee better access to inputs (proximity to the market, and higher income) and technical information (health and production) [8]. Marketing chains can be used as tracers of the livestock distribution, which is essential for the regulation of animal movements and animal trafficking. Tracing the livestock may help prevent and control the transmission of zoonotic diseases, especially endemic zoonotic diseases [9], such as Crimean-Congo hemorrhagic fever, Q fever, and brucellosis, which occur in some Eurasian countries such as Turkey, Pakistan, Afghanistan, and Iran [9,10]. The preference for livestock types, marketing time, and marketplace is also crucial for animal genetic resource and welfare management [8,11].

This study observed roadside livestock traders in urban and peri-urban areas in Yogyakarta, Indonesia, to identify both their individual profiles and business activities. To this end, this study focused on the procurements, sales, and patterns of marketing of the sacrificial livestock during the Eid al-Adha period to investigate buyers’ preferences for the origin of the animals, the place and time of livestock sales, and the reasons for selling livestock during the Eid al-Adha period. In addition, this study investigated the number of livestock that are offered and sold, as well as the average weight and price of livestock.

## Materials and Methods

### Ethical approval

The approval to conduct this study was obtained from the National Unity and Political Yogyakarta Province with the approval number 074/8900/Kesbangpol/2018 and a letter of recommendation from the Dean of the Faculty of Veterinary Medicine of Universitas Gadjah Mada, with the letter-number 295/Sains-Vet/VIII/2018.

### Study area

Yogyakarta is a province in Indonesia, which lies between 7^o^.33’-8^o^.12’ South latitude and 110^o^.00’-110^o^.50’ East longitude, with an area of 3185.80 km^2^, equal to 0.17% of the whole country (1,860,359.67 km^2^). Yogyakarta consists of four regencies (Kulonprogo, Bantul, Gunungkidul, and Sleman Regency) and one city (Yogyakarta city) [12]. The sample is divided into urban (Yogyakarta city) and peri-urban (Sleman and Bantul Regency) areas. Peri-urban areas are located between urban and rural areas.

### In-depth interviews of roadside livestock traders

In-depth interviews were conducted on a total of 36 roadside livestock traders in Yogyakarta, Indonesia: Twenty roadside traders in urban areas (Yogyakarta city) and 16 in peri-urban (Sleman and Bantul Regency) areas. The sampling was carried out by purposive sampling method. All interviews were conducted in the 2 weeks before Eid al-Adha celebration (August 5-23, 2018). The interviewed roadside traders were owners or members of an established stall. The collected data included roadside traders’ demographic profiles, preferences for buying and selling livestock, reasons for selling livestock in the Eid al-Adha period, prices, and the number of animals offered and sold during Eid al-Adha.

### Statistical analysis

The qualitative answers obtained in the interviews were transformed into categorical variables, and descriptive data analysis was conducted on numerical and categorical values. The data were analyzed using descriptive analysis (percentage and rank), and statistical analysis was performed using one-way analysis of variance on Statistical Package for the Social Sciences version 25 (IBM, USA) and used locations (urban and peri-urban areas) as factors. An average score (index) to rank the preferences was calculated with a formula adapted from Ojango *et al*. [13] with some modifications. The score obtained by the respondents’ answers was assigned points ranging from the most important (value=1) to the least important (value=2, 3, 4, 5). Furthermore, the total score was calculated as one divided by the value obtained by each answer, and all answered were summed. The index was obtained by dividing the total score for each answer by the total score of all the answers for every parameter. The ranking was determined by assigning the number “1” to major indices, followed by number 2, 3, 4, or 5 for smaller indices.

## Results

### Roadside livestock traders’ profile on Eid al-Adha festival

The roadside livestock traders’ demographics are presented in Table-1. The age, number of family members, trading experience, and number of employees are not significantly different in the two locations (urban and peri-urban areas). The trading time for the roadside traders in urban areas is longer (p<0.05) than in peri-urban areas. Most traders in urban areas are entrepreneurs, while they mostly are livestock traders in peri-urban areas. Most roadside traders are seasonal livestock traders and only open their stalls during Eid al-Adha. Most roadside traders in peri-urban areas open individual stalls and hire employees to help them. Most roadside traders in both urban and peri-urban areas sell sheep and goats, and no traders only sell cattle.

**Table 1:** Demographic profile of roadside livestock traders.

Parameters	Urban	Peri-urban	Pooled
Age (years)	43.53^a^±10.65	44.80^a^±15.09	44.13±12.72
Number of family members (head)	3.64^a^±1.50	4.45^a^±1.13	4.00±1.38
Trading experience (years)	17.41^a^±11.94	16.87^a^±14.33	17.18±12.85
Duration of trading (h/day)	17.82^b^±8.79	10.18^a^±7.39	14.00±8.83
Number of employees	4.70^a^±3.30	5.38^a^±4.96	5.00±4.00
Last education (%)			
Unfinished elementary school	6.30	25.00	14.30
Elementary school	6.30	8.30	7.10
Junior high school	31.30	16.70	25.00
Senior high school	43.80	33.30	39.30
College	12.50	16.70	14.30
The main job (%)			
Farmer	0.00	12.50	6.25
Livestock trader	25.00	37.50	31.25
Entrepreneur	37.50	25.00	31.25
Civil servants/retirees	6.25	0.00	3.13
Laborer	12.50	0.00	6.25
College student	0.00	12.50	6.25
Others	18.75	12.50	15.63
Trader’s scale (%)			
Large trader	5.26	6.25	5.71
Small trader	36.84	25.00	31.43
Opportunist trader	57.89	81.25	62.86
Stall ownership (%)			
Individual	50.00	62.50	55.60
Group	50.00	37.50	44.40
Kinds of livestock offered (%)			
Sheep only	21.10	25.00	22.90
Goats only	0.00	12.50	5.70
Cattle only	0.00	0.00	0.00
Sheep and goats	57.90	31.30	45.70
Sheep and cattle	5.30	0.00	2.90
Goats and cattle	0.00	6.30	2.90
Sheep, goats, and cattle	15.80	25.00	20.00

a,b Different superscripts on the same line indicates significant differences (p<0.05)

### Dynamics of buyer and livestock prices during Eid al-Adha period

The results of this study indicate that the purchasing days, the number of purchases (by the buyer), and the price dynamics in the Eid al-Adha period are not significantly different in urban and peri-urban areas in Yogyakarta (Table-2). Roadside traders buy livestock and begin to open their stalls on the 22^nd^ day before the Eid al-Adha festival. The livestock purchased by local people increases in the days leading up to the festival. Between the stall opening day and the 14^th^ day before the festival, only a few buyers visit the stalls, but the numbers of the buyers increase day by day. The majority of buyers visit the stalls on the 5^th^ day before the festival. The market prices are expressed in Indonesian Rupiah (IDR) (USD 1 = IDR 14,500 in August 2018). The prices of livestock (sheep and goats) at the market to increase on the 17^th^ day before the festival and keep rising with additional prices starting from IDR 210,000-/head. The highest price is registered on the 3^rd^ day before the festival, with a price difference of around IDR 224,800-/head of sheep or goat compared to a regular day. Prices begin to fall on the 3^rd^ day after the Eid al-Adha celebration, with a price drop up to IDR 473,300-/head of sheep or goat.

**Table 2:** Procurement, buyers, and prices of sacrificial livestock (sheep and goats) at roadside livestock trading during the Eid al-Adha period.

Parameters	Urban	Peri-urban	Pooled
		
	Mean±SE	Mean±SE	Mean±SE
Procurement of sacrificial livestock			
Starting from (days before the festival)^NS^	23.13±5.57	21.38±6.06	22.34±4.03
Until (days before the festival)^NS^	6.42±3.01	3.83±1.58	5.56±2.06
Livestock buyers			
Most (days before the festival)^NS^	4.50±1.02	5.70±1.68	5.05±0.93
Fewest (days before the festival)^NS^	12.00±5.05	17.29±2.34	14.64±2.77
Prices of livestock at the market			
Start to go up (days before the festival)^NS^	18.67±4.34	16.78±3.67	17.96±2.99
Highest (days before the festival)^NS^	2.71±0.64	3.13±0.13	2.93±0.30
Start to go down (days before the festival)^NS^	2.11±0.39	2.50±0.57	2.29±0.33
Increase by (IDR×10^6^/head)^NS^	0.17±0.06	0.26±0.11	0.21±0.06
Highest (add IDR×10^6^/head)^NS^	0.23±0.09	0.25±0.18	0.23±0.08
Decreased by (IDR×10^6^/head)^NS^	0.68±0.27	0.33±0.09	0.47±0.12

NS Nonsignificant. IDR=Indonesian Rupiah

### Roadside traders’ preference with the momentum of Eid al-Adha festival

Roadside livestock traders in both urban and peri-urban areas buy livestock at the animal markets. The Eid al-Adha period is a strategic moment for selling livestock due to the high prices. Most customers who buy livestock in both urban and peri-urban areas are end-consumers who directly buy from the stalls (Table-3). Most roadside traders do not practice different prices to different consumers. Some argue that a price difference of IDR 170,000- and IDR 210,000- exists in urban and peri-urban areas, respectively. According to most roadside traders, significant differences exist in livestock prices between Eid al-Adha and ordinary days, and the price in urban areas is slightly higher. Hence, traders benefit from the Eid al-Adha moment (Table-4).

**Table 3:** Ranking analysis of preferences for livestock purchased and sold, sales moment, and reasons for sales during the Eid al-Adha period.

Parameters	Urban		Peri-urban		Pooled	
		
	Index	Rank	Index	Rank	Index	Rank
Livestock purchased from						
Smallholder farmers	0.30	2	0.43	2	0.39	2
Livestock trader	0.11	3	0.04	4	0.08	4
Village collectors	0.02	5	0.04	4	0.03	5
Animal markets	0.48	1	0.26	1	0.39	1
Organizations/groups	0.11	3	0.08	3	0.10	3
Livestock sold during						
Eid al-Adha period	0.38	1	0.68	1	0.51	1
Ordinary days	0.25	2	0.10	3	0.19	2
*Aqiqah*	0.23	3	0.13	2	0.19	2
Market days	0.11	4	0.05	4	0.08	4
Others	0.03	5	0.04	5	0.04	5
Livestock sold to						
Consumers/individuals	0.68	1	0.45	1	0.55	1
Village collectors	0.02	3	0.04	5	0.03	4
Animal markets	0.13	4	0.12	3	0.13	3
Slaughterhouse	0.00	-	0.06	4	0.03	4
Organization/groups	0.17	2	0.34	2	0.26	2
Reasons to sell during Eid al-Adha period						
Higher price	0.37	1	0.37	1	0.37	1
Easy to sell	0.35	2	0.16	4	0.26	2
Many buyers	0.27	3	0.21	3	0.24	3
Others	0.02	4	0.26	2	0.13	4

1,2,3,4,5: Ranking of the smallest value is a priority

**Table 4:** Preference of roadside livestock traders on selling their livestock during the Eid al-Adha period.

Parameters	Urban	Peri-urban	Pooled
Difference in selling price to different consumers/selling place (%)			
Yes	42.86	38.46	40.74
No	57.14	61.64	59.26
The average of price difference on different consumers/selling place (IDR)	170,000	210,000	183,333
Difference in selling prices during Eid al-Adha and ordinary days (%)			
Yes	94.12	80.00	87.06
No	0.00	0.00	0.00
Do not know	5.88	20.00	12.94
Difference in price of livestock during Eid al-Adha and ordinary days (IDR)			
Sheep	841,538	831,923	837,692
Goats	801,000	700,714	759,701
Cattle	5,882,143	5,700,000	5,815,909
Is the sales number during Eid al-Adha satisfying? (%)			
Yes	80.00	75.00	77.50
Neutral	20.00	25.00	22.50
No	0.00	0.00	0.00

IDR=Indonesian Rupiah

### Livestock offered and sold during Eid al-Adha period

No significant differences exist in the livestock offered and sold, average body weight, and prices between urban and peri-urban roadside traders during the Eid al-Adha period (Table-5). The highest number of livestock offered is sheep. The average body weight of sheep and goats for sale is similar, around 29.6 kg, and sheep is more expensive, around IDR 200,000-/head. The cattle sold weigh around 283 kg, with a selling price of IDR 19,386,000-/head. Almost all offered livestock are sold.

**Table 5:** Average number and price of livestock sold by roadside livestock traders during Eid al-Adha festival.

Parameters	Livestock	Urban	Peri-urban	Pooled
		
		Mean±SE	Mean±SE	Mean±SE
Livestock offered (head)^NS^	Sheep	65.77±11.74	67.69±18.93	66.21±9.96
	Goat	21.11±7.07	33.20±9.02	25.43±5.59
	Cattle	5.73±1.67	3.40±0.51	5.00±1.17
Livestock sold (head)^NS^	Sheep	60.16±9.54	59.86±12.53	60.05±7.53
	Goat	20.11±7.23	32.00±9.72	24.36±5.79
	Cattle	4.55±1.66	3.25±0.63	4.20±1.22
Body weight (kg)^NS^	Sheep	30.36±1.51	27.25±1.06	29.61±1.19
	Goat	30.13±2.36	28.75±4.27	29.67±2.01
	Cattle	315.25±14.59	250.00±45.64	282.63±25.38
Price (IDR×10^6^)^NS^	Sheep	2.81±0.20	2.77±0.22	2.79±0.20
	Goat	2.67±0.42	2.34±0.53	2.53±0.32
	Cattle	19.61±1.03	19.00±1.16	19.39±0.75

NS Non-significant. IDR=Indonesian Rupiah

## Discussion

### Roadside livestock traders’ profile on Eid al-Adha festival

Traditional slaughtering and sacrifice of livestock, generally sheep and goats, for festivals and religious holiday play an essential role in Muslim countries [4]. Apart from the Eid al-Adha period, most consumers in Turkey do not directly buy animals from an animal market or a farm. They generally buy meat in smaller quantities from local butchers and supermarkets. Either consumers’ attitude or modern urban life, which has limited ties with rural areas, motivates this choice. However, it is necessary to resort to experts’ opinion in the selection of sacrificial animals [14]. The situation is different in Indonesia, where, on Eid al-Adha, most consumers directly buy from farmers, animal markets, and stalls offering sacrificial livestock. Both in urban and peri-urban areas, during the Eid al-Adha period, people open tents to sell sacrificial livestock. Ahead of the Eid al-Adha celebration, many roadside traders sell livestock on the roadside to take advantage of this moment. As mentioned above, the time spent to trade the livestock is longer in urban areas, where most stalls are open on rented land, at the edge of the city. In addition, the stalls stay open for longer because the number of buyers or people passing by the road is very high.

In this study’s sample, roadside traders consist of 31.43% regular traders and 68.57% opportunist traders, who only open their stalls during the Eid al-Adha period, both individually and in groups. Their characteristics are similar to those of the livestock traders in Tel Sheva, Southern Israel, where three types of livestock traders exist [4], namely, large, small, and opportunist livestock traders. Large livestock traders handle more than 200 animals a year, trade all year, and do not attend weekly markets, but the sellers and buyers visit their farms. Small livestock traders handle up to about 20 animals, trade with partners, and attend weekly markets. Opportunist livestock traders handle livestock sporadically when they feel that a profit can be made. Roadside livestock traders in Yogyakarta are mostly small and opportunist livestock traders. Large livestock traders in this area usually run their business on their farm, and their customers visit them to buy and resell the livestock.

The roadside traders build tents on the roadside, where the buyers can easily find them. The livestock sold are sheep, goats, and cattle, in line with other Muslim countries, such as in Pakistan, where cattle, buffalo, sheep, goats, and camels are offered during the 3 days of Eid al-Adha festival. As Eid al-Adha approaches, the craze of buying animals for sacrifice intensifies. Streets, lanes, and alleys are filled by the sacrificial animals. Roadsides are filled with makeshift tents to recover the livestock [2].

### Dynamics of buyer and livestock prices during Eid al-Adha period

The prices applied by the roadside traders in urban and peri-urban areas are similar. Stalls open 3 weeks before the festival, but most buyers visit the stalls the last week before the festival. The closer to the festival, the higher the price, and prices drop on the 2^nd^ day after the festival. This pattern is in line with other Muslim countries such as Turkey, where a sacrificial animal market is established in many provinces and cities once a year (20-30 days before the Feast of Sacrifice). The market is settled for a few weeks, and sellers from different regions come to sell their animals. Customers repeatedly visit the market until the 1^st^ day of the feast to buy sacrificial animals at the best price [14]. According to the roadside traders, the prices of livestock for sacrifice depend on the breed, physical conditions, and distinctive characteristics demanded by the community. For instance, the prices of livestock in India [15] are based on physical condition and market demand. Physical conditions include the health of the animal, physical configuration, and average weight (depending on the age), among the others.

Toward the Eid al-Adha festival, the demand for livestock in various regions increases rapidly [16]. Urban traders (for sheep and goats) sell the livestock at market prices, which can fluctuate considerably. Prices of rams and bucks markedly increase before Eid al-Adha due to the high demand for animals to be sacrificed [4]. Meat consumption is related to religious and cultural traditions. Some ethnicities, including Muslims, have the highest demand for meat [17], especially during the Eid al-Adha festival. As Eid al-Adha approaches, the demand for livestock and its feed increases, along with the increasing price of feed [18].

### Roadside livestock traders’ preference with the momentum of Eid al-Adha festival

This study conducted a ranking analysis to identify the preferences of roadside livestock traders on procurement, selling time, and a reason for selling livestock during the Eid al-Adha period. The animal market is the primary place for livestock procurement because many breeds and price options exist. Farmers usually sell their livestock individually. Village collectors are the last choice to buy since their prices are relatively high compared to other sellers. Several considerations are made before purchasing the livestock, namely, ease in receiving the livestock, transporting cost, prices, and distance to the market [7].

Roadside livestock traders sell their livestock during Eid al-Adha to benefit from the high price, the ease to sell the livestock, and the number of potential buyers. Those who trade as their primary occupation also sell livestock for *aqiqah* and on ordinary days. The high demand for sheep and goats to sacrifice is a big business opportunity, and both farmers and traders adapt to the requirements for livestock sacrifice (*kurban*) [16].

In general, Muslims in Eurasia slaughter animals by themselves, but someone else can be appointed to undertake the slaughter on their behalf [10]. In Indonesia, especially in Yogyakarta, sacrificial livestock is mostly bought from farmers, the animal markets, and roadside traders. Then, the buyer hands over the livestock to the *kurban* committee in the mosque to be slaughtered and distributed by the committee. They rarely slaughter their sacrifice livestock and distribute it by themselves. This is local wisdom to build togetherness among citizens.

Roadside livestock traders mostly sell their livestock during Eid al-Adha to individual consumers who visit their stalls (Table-4). They also receive orders from orphanages, schools, and other organizations or institutions. Half of them do not practice different prices among their customers. Some roadside livestock traders apply different prices to consumers who directly bid with the ordering organization, with a price difference of around IDR 183,333. In Indonesia, sheep are usually sold by local traders in rural markets [19]. Local traders usually pay for livestock to the farmers, and the prices are agreed on at the place of trading. Most farmers prefer this way. The diversity of consumers requires the maintenance of genetic diversity [8] so that distribution channels can provide a variety of animals based on consumers’ needs.

In the Eid al-Adha period, economic activity increases. The selling price of livestock increases on average by 35%, around IDR 837,692,-/head for sheep, IDR 759,701,-/head for goats, and IDR 827,692,-/head for cattle (Table-4). The feast of Eid al-Adha is associated with a positive and highly significant difference in price, compared with other periods, in Burkina Faso, West Africa [8]. During the Eid al-Adha period, in Yogyakarta, traders witness an increase in the selling price of livestock between36.57% and 71.77% [7]. In Pakistan, the Eid season increases economic activity around the country. With the cattle price ranging from Rs. 80,000 to Rs. 5,000,000 and goats from Rs. 25,000 to Rs. 500,000, the market does not offer a wide range of animals to customers characterized by different livelihood levels [2].

Most roadside traders (77.50%) are satisfied with their business in the Eid al-Adha period. However, 22.50% of the roadside traders are mediocre satisfied because they mainly are livestock traders. Most roadside traders benefit from the increase in prices during this period. The results of this study confirm the findings of previous research that livestock prices increase during Eid al-Adha, thus allowing farmers to make a higher profit [20]. Most consumers think that *kurban* market contributes to the country’s economy and prefers to buy sacrificial animals from these markets rather than local farms [14].

### Livestock offered and sold during Eid al-Adha period

During the Eid al-Adha celebration, Muslims across the world present an animal as a sacrifice. The animal must either be cattle, sheep, goat, buffalo, or camel with specific requirements such as not pregnant and in good condition [21]. The animal has to “be perfect,” that is, it must not be cut, castrated, have docked tails or a broken horn, be lame or damaged. The minimum age for sheep is 6 months old and 1 year old for goats [4,22]. In this study’s sample, from a total of 20 and 16 roadside traders in urban and peri-urban areas, respectively, 95.00% and 81.25% of traders sell sheep, 70.00% and 75.00% goats, and 20.00% and 31.25% cattle.

The highest number of livestock offered is sheep. The average body weight of sheep and goats is similar, around 29.6 kg, with a higher price for sheep, with a difference of around IDR 200,000-/head. Cattle have an average body weight of around 283 kg, with an average price of IDR 19,386,000-/head. Almost all livestock offered are sold. The proportion of sheep offered and sold is higher than that of goats or cattle because the Muslims community in Yogyakarta prefers to sacrifice sheep on Eid al-Adha celebration [7]. Higher supply of and demand for sheep are recorded during religious festival due to the higher religious value of sheep and religious-dependent consumption patterns [23].

Indonesia is a country where small ruminants play an essential role in the livelihoods of rural people and religious festivals [7,24], especially sheep [25,26]. Sheep are widely used by Muslims for sacrificial rituals on Eid al-Adha festival, *aqiqah*, and salvation events [19,27]. Muslim families who have a high standard of living will slaughter sheep or goats during Eid al-Adha festival [7].

The livestock sacrifice choice is a fundamental part of the preparation of the ritual mentioned above, and it is a part of the ritual itself. The decision is driven by three factors: The animals, the buyers, and the sellers. In choosing an animal for *kurban*, people have several motivations, such as financial opportunity, choosing a less tiring practice, harmony among partners, cultural preference, risk aversion, and distributing more meat [14]. The number of animals slaughtered for Eid-Al Adha is much higher compared to the number of animals slaughtered daily [1,28]. The amount of sheep sold in the Eid al-Adha period is 127% higher than in the ordinary period, with the number of livestock sold being 182% higher than in regular periods. The price and body weight of sheep also increase by 69% and 35%, respectively [6]. In America, at this festival, the weight of the sheep in demand is 60-80 pound, and yearling goats or large goatlings are 60-100 pound [17].

Targeting specific consumer groups such as Muslim consumers may be a viable alternative to increase income in the small ruminant sector [21]. Meat demand is tied to religious and cultural conditions, which vary across time and result in district preferences for specific products. Producers may want to consider these holidays as they plan the breeding and marketing schedule [17] as they may benefit from higher prices during the Eid al-Adha festival. Farmer and traders need to plan their production and supply to match the fluctuating but predictable patterns of demand [23]. The different markets represent district level demands for genetic characteristics, entailing interesting consequences for animal genetic resource management. Any breeding programmed should take this diversity into account to allow this sector to contribute to the sustainable development of the country [8].

## Conclusion

The characteristics of roadside livestock traders, who market livestock during Eid al-Adha period in urban and peri-urban areas in Yogyakarta, Indonesia, are similar. Sheep are more desirable than goats and cattle during this period. Eid al-Adha period assures a higher profit to the roadside livestock traders in both urban and peri-urban areas.

### Authors’ Contributions

WTA supervised the present study, and IGSB and RW designed and coordinated the study. AI performed the experiment, analyzed the data, and wrote the manuscript. The final manuscript has been read and developed in consultation with all authors. All authors read and approved the final manuscript.
